# Wild chimpanzees’ use of single and combined vocal and gestural signals

**DOI:** 10.1007/s00265-017-2325-1

**Published:** 2017-05-27

**Authors:** C. Hobaiter, R. W. Byrne, K. Zuberbühler

**Affiliations:** 1 School of Psychology and Neuroscience, University of St Andrews, St Marys College, South Street, St Andrews, KY16 9JP Scotland; 2 Budongo Conservation Field Station, Masindi, Uganda; 3 Department of Comparative Cognition, University of Neuchatel, Neuchâtel, Switzerland

**Keywords:** Multimodal, Ape, Language origins, *Pan troglodytes*, Signal combination

## Abstract

**Abstract:**

We describe the individual and combined use of vocalizations and gestures in wild chimpanzees. The rate of gesturing peaked in infancy and, with the exception of the alpha male, decreased again in older age groups, while vocal signals showed the opposite pattern. Although gesture-vocal combinations were relatively rare, they were consistently found in all age groups, especially during affiliative and agonistic interactions. Within behavioural contexts rank (excluding alpha-rank) had no effect on the rate of male chimpanzees’ use of vocal or gestural signals and only a small effect on their use of combination signals. The alpha male was an outlier, however, both as a prolific user of gestures and recipient of high levels of vocal and gesture-vocal signals. Persistence in signal use varied with signal type: chimpanzees persisted in use of gestures and gesture-vocal combinations after failure, but where their vocal signals failed they tended to add gestural signals to produce gesture-vocal combinations. Overall, chimpanzees employed signals with a sensitivity to the public/private nature of information, by adjusting their use of signal types according to social context and by taking into account potential out-of-sight audiences. We discuss these findings in relation to the various socio-ecological challenges that chimpanzees are exposed to in their natural forest habitats and the current discussion of multimodal communication in great apes.

**Significance statement:**

All animal communication combines different types of signals, including vocalizations, facial expressions, and gestures. However, the study of primate communication has typically focused on the use of signal types in isolation. As a result, we know little on how primates use the full repertoire of signals available to them. Here we present a systematic study on the individual and combined use of gestures and vocalizations in wild chimpanzees. We find that gesturing peaks in infancy and decreases in older age, while vocal signals show the opposite distribution, and patterns of persistence after failure suggest that gestural and vocal signals may encode different types of information. Overall, chimpanzees employed signals with a sensitivity to the public/private nature of information, by adjusting their use of signal types according to social context and by taking into account potential out-of-sight audiences.

**Electronic supplementary material:**

The online version of this article (doi:10.1007/s00265-017-2325-1) contains supplementary material, which is available to authorized users.

## Introduction

One means to explore the evolutionary origins of human communication is to examine primate communication for areas of overlap and disjunction, in particular among great apes (Fitch 2010). Human and non-human animals share the ability to communicate using different types of signals, including vocalizations, gestures, facial expressions, and body movements (e.g. apes: Goodall 1968; van Hooff 1973; de Waal 1988; spiders: Uetz and Roberts 2002; frogs: de Luna et al. 2010; birds: Cooper and Goller 2004). In human communication, the integration of information from vocalizations and gestures is a universal feature. For example, across cultures, parents incorporate gestures into their infant-directed speech (Gogate et al. 2006) and gestures add to and may even contradict the information provided in adult speech (Goldin-Meadow 1999). Indeed, humans communicate by combining signals from speech, gesture, facial expression and body movements into a single stream (Iverson and Goldin-Meadow 1998), yet the study of communication in non-human primates has traditionally focused on vocal, facial or gestural signals in isolation.

Following a seminal paper by Partan and Marler (1999), a number of researchers took up the challenge of investigating ‘multimodality’ in non-human primate communication (Partan 2002; Palagi and Norscia 2008; Micheletta et al. 2012; Higham et al. 2013; Rigaill et al. 2013; Wilke et al. 2017). In great apes, this was largely done with captive groups (Leavens and Hopkins 2005; Pollick and de Waal 2007; Pollick et al. 2008; Leavens et al. 2010; Genty et al. 2014). One common conclusion from this research has been that ‘multimodal’ signals in primates evolved not to enlarge the range of information that could be communicated, but simply to enhance detection (Pollick and de Waal 2007; Leavens et al. 2010; Micheletta et al. 2012; although c.f. Genty et al. 2014 for some evidence of additional communicative cues provided by bonobo vocal-gestural combinations), a hypothesis further supported by neurobiological data (Taglialatela et al. 2011).

Here, however, it is necessary to make a distinction that has not been clear in the primate communication literature. The integration of different sources of information can occur (a) within a single type of signal: for example, human speech is itself multisensory, involving a combination of visual and auditory cues in both production and perception (Sekiyama and Tohkura 1993; Schwartz et al. 2004). Similarly, a chimpanzee ‘pant-hoot’ vocalization provides striking visual as well as auditory cues, while the majority of chimpanzees’ gestures contain distinctive auditory cues to which gestures are being produced even if the signaller is out-of-sight, e.g. an *Object shake* versus *Hit object*. Audible gestures typically incorporate an object, including the ground or another individual, in order to generate distinctive sounds. In addition, information can be integrated (b) through the combination of different signal types, as in the use of speech-accompanying gestures in humans (Goldin-Meadow 1999), or the combination of a gesture and facial expression in human and non-human primates (Parr et al. 2007). The term ‘multimodal signalling’, as it has been used in some animal communication studies, refers to the *combination of information from different sensory channels* (visual, auditory, tactile, olfactory) and in this sense many individual primate signals are inherently multimodal (Partan and Marler 1999). In each case, the particular combination of cues available to the recipient depends on their attentional state and the perceptual range of their individual senses. Thus, in chimpanzees, the single gestural signal *Object shake* contains both visual and acoustic information; an individual in sight of the signaller receives both modalities, whereas an out-of-sight individual receives only acoustic information. Within the primate literature, some researchers have adopted this definition (e.g. Partan 2002; Palagi and Norscia 2008; Micheletta et al. 2012; Rigaill et al. 2013). However, other researchers have used the term multimodal signalling to refer to the combination of *different signal types*, such as a gesture, vocalization, or facial expression (Pollick and de Waal 2007; Liebal et al. 2013; Genty et al. 2014; Wilke et al. 2017). Here, a silent visual facial expression, such as a *Bare-teeth grin*, could be combined with a silent visual gesture, such as a *Reach*: this would be considered multimodal, despite transmitting information through only a single sensory modality. Here, we employ the wider standard definition of multimodal signalling (e.g. Partan and Marler 1999), treating the combination of gestures and vocalizations or facial expressions as ‘signal combinations’.

Signal combinations are of particular interest for understanding great ape cognition, given the widespread intentional use of gestures (e.g. Tomasello et al. 1985; Genty et al. 2009) and specific vocalizations (e.g. Schel et al. 2013a; and see Townsend et al. 2016), and a documented ability to both suppress (Brosnan and de Waal 2002; Townsend et al. 2008) and produce vocalizations to favour specific audiences (Crockford et al. 2012; Schel et al. 2013a). Whereas the multimodal components of a single signal are fixed by its production (a chimpanzee cannot produce a pant-hoot sound without also moving its lips), signal combinations permit flexibility, such as the transmission of acoustic information together with a silent visual signal, potentially offering additional levels of sophistication to the communicative repertoire. For example, both vocalizations and gestures contain audible information about the signaller, for example that they are in the presence of food (Schel et al. 2013b) or danger (Schel et al. 2013a), or that they desire to be groomed, to threaten or to travel (Hobaiter and Byrne 2014). However, with the exception of drumming (Arcadi et al. 1998), there is no evidence that the audible component of gestures encodes the signaller’s identity, whereas a range of vocalizations contain information about the identity or group membership of the signaller (Crockford et al. 2004) that other chimpanzees are capable of recognizing (Herbinger et al. 2009; Kojima et al. 2003). Thus, one consequence of a signaller employing a vocal rather than a gestural signal, or adding a vocal signal to a gestural one, is that they reveal their identity not only to any intended immediate recipient of the visual information in the signal, but also to other, potentially out-of-sight individuals or eavesdroppers.

Conversely, while on consortship, wild chimpanzees use gestures with limited audibility. In other circumstances, chimpanzees typically combine gestures that are audible over a short- and long distances within the same communicative sequence. However, during consortship, they restrict signal use to gestures containing visual, tactile and short-distance-only audible cues (for example, *Object shake*, but not *Object drum*; Hobaiter and Byrne 2012). By doing so, they appear to take into account the perceptual variability of two different audiences: the female in close proximity to them, and the other males who are out of visual and ‘short-range’ audible information.

Almost nothing is known on how wild chimpanzees employ the full repertoire of signals available to them. A wide range of variables have been shown to impact chimpanzee gestural and vocal communication, for example: age (Tomasello et al. 1985; Hayaki 1990; Kojima 2008; Hobaiter and Byrne 2011a, b); sex (Laporte and Zuberbühler 2010; Frohlich et al. 2016); rank (Clark and Wrangham 1993; Schel et al. 2013b; Bard et al. 2014); and social bonds (Crockford et al. 2012; Fedurek et al. 2013). We present the results of a systematic study of the individual and combined use of vocal and gestural signals in wild great apes, the Sonso chimpanzee community of Budongo Forest, Uganda. We begin by providing a detailed description of the single and combined use of vocal and gestural signals as naturally produced by signallers and perceived by recipients of different age-sex classes and of different ranks. We then investigate possible explanations for the selective use of gestures, or vocalizations, or combinations of both.

### One: redundancy versus refinement

While the type of cues present in vocalizations are fixed (visual and audible), gestures may be produced with a range of different cues (for example, silent visual vs. tactile). The combination of vocal and gestural signals might allow signallers to produce communication adapted to a particular environment or goal. The addition of a different modality of cue could simply provide a ‘backup’ channel ensuring that the signal is received (Partan and Marler 2005; fowl: Smith and Evans 2008; wolf spiders: Uetz and Roberts 2002); alternatively, the different cues could provide different types of information allowing for refinement or increased specificity of the signal content (Partan and Marler 2005; starlings: Jacob et al. 2011; bonobos: Genty et al. 2014). We examine the use of individual and combined signals, following the failure of an initial signal, for evidence of redundancy or refinement. If chimpanzees combine gestural and vocal signals as a form of redundancy, we predict that signal combinations will be produced more frequently following the failure of an initial single signal. If chimpanzees combine gestural and vocal signals to refine the content of the signal, we predict that the use of single or combined signals will be independent of the failure of an initial signal.

### Two: private versus public messaging

Signals are not transmitted in isolation, whether targeted to a particular recipient or broadcast widely; the identity and social context (such as feeding, travelling or agonism) of both signaller and audience impact signal use in chimpanzees (Mitani and Nishida 1993; Slocombe and Zuberbühler 2007; Hobaiter and Byrne 2014) as they do in many species (e.g. Siamese fighting fish: Doutrelant et al. 2001; pied babblers: Engesser et al. 2016; African elephants: Soltis et al. 2005; see Smith 1965). Different cues may be detected by different audiences. While visual and tactile cues are typically limited to a known audience, signals that incorporate audible cues may be received by an audience that is out-of-sight; such variation allows for signallers to potentially limit cues to a particular audience, avoiding possible costs of eavesdropping (Higham and Hebets 2013). If chimpanzees are sensitive to the public/private message distinction, we predict that when costs of eavesdropping are high, signallers will use gestural rather than vocal signals; conversely, when conveying signaller identity is beneficial, we predict the use of vocal rather than gestural signals, thus reliably revealing information on signaller identity.

## Method

### Study site and subjects

The study was conducted at the Budongo Conservation Field Station (BCFS), located in the Budongo Forest Reserve, Uganda. The reserve includes 482 km^2^ of continuous medium-altitude, semi-deciduous, secondary-rainforest growth (Eggeling 1947). At the start of data collection in January 2012, the Sonso study community of chimpanzees consisted of 72 named individuals. Following Reynolds (2005), we defined age groups as follows: infants (0–4 years), juveniles (5–9 years), subadults (m: 10–15 years, f: 10–14 years) and adults (m: 16+ years, f: 15+ years). Using these categories, the initial group composition was 31 adults (10 males and 21 females), 14 subadults (4 males and 10 females), 17 juveniles (4 males and 13 females) and 9 infants (4 males and 5 females).

### Data collection

We used focal animal sampling (Altmann 1974) with 1-h focal follows during 3 months from January to March 2012. It was not possible to record data blind because our study involved focal animals in the field. The Budongo forest is a dense secondary rainforest with thick ground cover, which makes continuous observations a challenge. If a focal individual was out of sight for less than 10 min, we interrupted data collection and resumed it once the individual was back in sight. If a focal individual was out of sight for more than 10 min, the focal follow was terminated. On occasion, it was necessary to accumulate two shorter focal follows to obtain the required 1-h criterion. No single focal follow was shorter than 20 min and, on a given day, only one focal sample was collected within a single time period or from adjacent time periods (e.g. morning and mid-day, or mid-day and afternoon, see “Definitions”: Activity).

Data for each series of signals included the following: date, time, location, party size, activity of the focal individual, the role of the focal individual (signaller or potential recipient), number of signals, the mode of signalling (gestural, vocal, gesture-vocal combination), the type of vocalizations or gestures, whether the signals appeared to be directed to the focal individual (produced in the direction of the focal and accompanied by signs of visual attention from the signaller, such as looking at or checking back to the focal). Where possible, we recorded the identity of the signaller (when the focal was a potential recipient), and the distance of the signaller or potential recipient. Party size is collected on a 15-min interval basis as part of the BCFS long-term data by a permanent staff of field assistants. The party includes individuals within a 35-m radius of the focal individual, which represents the average travel spread of parties in Budongo (Newton Fisher 2004). The distance was estimated and party composition recorded by both CH and her field assistant.

We recorded detailed data on a single focal individual. Where our focal emitted a signal, they were defined as a *signaller*. In gestural research, a recipient is usually defined as an individual to whom a signal is targeted with signallers frequently checking the recipient’s state of attention or monitoring the response (e.g. Hobaiter and Byrne 2011a, b). In vocal research, many vocalizations have evolved to communicate over long distances, suggesting that any individual within the range of the signal is a potential recipient. Here, we considered any focal individual as a *potential recipient* if (1) it was visibly targeted by a signaller, such as with a *Reach* gesture accompanied by response waiting or checking of their attention, or (2) it was exposed to a signal directed to another individual or simply broadcast to the wider group, such as a *Pant-hoot* produced while an individual climbs a tree 200 m away. In the second case, although not specifically targeted, focal animals could still extract information from the signal, such as changes in rank between males or the location of a feeding tree. We aimed to record facial expressions on an all occurrence basis, but observation conditions prevented systematic data collection, so they were not included in the analyses.

#### Age, sex, and male rank

We balanced the data set so that both sexes were equally represented within the adult and subadult categories (Table 1). This was not possible within the juvenile and infant categories as the 4th potential juvenile male and four of the nine infants belonged to peripheral females who are difficult to observe. Mature male chimpanzees (adult and subadult) were ranked in a linear fashion from 1 to 9 on the basis of unidirectional pant-grunt data collected as part of the long-term records.

**Table 1:** Number and type of signals recorded (production and exposure), age and sex for focal individuals. g = gesture, v = vocal, c = combination

Age class	Sex	ID	Obs (h)	Signalled			Exposed to		
				g	v	c	g	v	c
Alpha	Male	NK	6	77	48	8	57	566	43
Adult, *n* = 8	Male, *n* = 4	HW	3	2	10	3	12	129	5
		KT	3	6	34	1	21	116	14
		MS	3	11	18	6	24	172	4
		ZF	3	17	23	12	16	169	10
	Female, *n* = 4	JN	3	9	18	0	30	177	18
		ML	3	17	29	4	31	99	15
		NB	3	9	23	3	13	190	24
		OK	3	6	33	7	22	151	12
Subadult, *n* = 8	Male, *n* = 4	FK	3	15	24	4	21	110	5
		KZ	3	6	19	1	20	167	13
		PS	3	5	31	2	24	180	25
		ZD	3	4	15	0	35	271	26
	Female, *n* = 4	JT	3	13	15	0	26	178	11
		KA	3	2	21	1	10	128	7
		KR	3	11	6	1	40	253	26
		RS	3	7	11	1	46	205	20
Juvenile, *n* = 8	Male, *n* = 3	JM	3	3	9	1	37	144	9
		KC	3	10	21	9	35	232	26
		KS	3	8	18	2	28	125	14
	Female, *n* = 5	HY	3	5	8	1	63	184	11
		KB	3	10	15	2	52	219	17
		KX	3	6	4	1	15	67	2
		RM	3	7	3	0	29	163	11
		FA	3	9	26	4	15	153	12
Infant, *n* = 5	Male, *n* = 2	JB	3	23	7	0	36	145	13
		MB	3	42	18	5	33	111	10
	Female, *n* = 3	RF	3	7	27	2	54	182	21
		HE	3	28	7	0	56	151	4
		KH	3	13	6	1	67	194	12
Total	*n* = 30 (14 m, 16 f)		93	388	547	82	968	5331	440

### Definitions

We define a communicative *unit* as an individual call, gesture or facial expression produced once or repeatedly as part of a *series*. We use the term *signal* to refer to the production of either a single unit or series of units followed by a pause of >1 s (as per Hobaiter and Byrne 2011a, b, 2012). When calculating the number of units within a series, repetitions of the same type of unit were not counted as multiple units. We use the term ‘vocal’ for signals composed only of vocalizations, ‘gestural’ for tactile, silent visual and auditory gestures, and ‘combination’ for combinations of vocal and gestural units in a single signal.

We considered *persistence* to be the production of an additional signal after *response waiting* and employed this as an indication of the *failure* of the previous signal. Response waiting was defined as a pause of >1 s in which no new activity was started by the signaller in either solitary (e.g. self-grooming, feeding) or social situations (e.g. travelling, grooming). Failure was only scored for signals produced for the focal individual. We considered the start of a new activity to represent the end of a communication event.

As continuous filming of focal individuals was difficult, we dictated all observations into a handheld recorder. Our goal was to document all signals produced and received by the focal animal. We categorized units based on previously published repertories of wild chimpanzee, i.e. 66 gestures (Hobaiter and Byrne 2011a) and 15 vocalizations (Crockford and Boesch 2005).

#### Activity

Chimpanzee activity rates fluctuate throughout the day. In order to control for daytime effects, we balanced sample collection across three time periods. A total of 3 h of data were collected for each individual, 1 h in the morning (‘*am*’ = 7–10 am), 1 h over mid-day (‘*mid*’ = 10 am–1 pm) and 1 h in the afternoon (‘*pm*’ = 1–4 pm).

#### Dominance

The alpha male in a chimpanzee community occupies a unique position, which in itself may affect the communicative environment in which he exists. For example, all other individuals within the community pant-grunt to him, whereas he will not pant-grunt to any other individual. To control for alpha position as a source of possible variation, the alpha male’s data were not included in analyses of ‘adult male’ behaviour. As there can only be one alpha male in the community at any one time, we were unable to collect data from multiple individuals within this category. Variation in behaviour between communities may be affected by variation in both a community’s social structure (size, sex ratio) and its environment (Schöning et al. 2008). Alpha male tenures may last for over a decade, making repeated observations within a single community difficult. As we wished to limit error due to a small sample size, we collected a total of 6 h of data from the alpha male, 2 h per time period, and then calculated a mean rate per hour from this, to allow comparison with the other age/sex categories.

#### Context

Focal activity was recorded to the nearest minute. In order to control for possible effects of context on the signalling of the focal individual, for example during the investigation of male rank, we totalled the number of minutes of observation time in which the focal individual was recorded as having engaged in that activity at least once (see Table 2).

**Table 2:** Definition of the behavioural contexts for communication in wild chimpanzees. Examples of the signals recorded within each context are also provided; however, signals are not specific to an individual context and may occur across several of them

Context	Definition
Affiliation	One individual seeks social support or positive physical contact from another, for example: some greetings, when distressed or during reconciliation. Includes gestures such as *Touch* (e.g. *testicles*) or *Shake hands* and vocalizations such as *pant* or *pant-grunt*.
Agonism	One individual seeks to chase away or physically attack another; typically results in other individual leaving the party or engaging in physical fight. Signaller is typically piloerect. Includes gestures such as *Object shake* or *Slap* and vocalizations such as *bark* or *scream*.
Begging^a^	One individual requests food, or access to food, from another. Includes sitting and peering, gestures such as *Reach* or *Mouth stroke*, and vocalizations such as *whimper* or *pant*.
Consortship^b^	Includes individuals engaged in or soliciting for consortship behaviour (not observed during data collection period).
Displace^a^	One individual seeks to physically displace another, does not include running towards another or other individual running away (see display at or agonism). Includes gestures such as *Hand fling* or *Slap object*, and vocalizations such as *bark*.
Display at^c^	Social displays such as running through the group or towards another individuals. Includes gestures such as *Object shake* and or vocalizations such as *pant-hoot* or *pant-roar*.
Feeding	Primarily the location, preparation and ingestion of food, includes nursing, and drinking. Includes hunting behaviour post-kill, i.e. meat-eating. Gestural signals rare but include, for example, *Arm raise*, and vocalizations such as *food-grunt* or *pant-hoot*.
Grooming	An individual participates in grooming or requests grooming from another. Includes gestures such as *Directed push* or *Big loud scratch*, and vocalizations such as *pant* or *pant-grunt*.
Hunting^a^	Includes patrolling (individuals walk one behind each other in a line while remaining silent and highly vigilant), chasing and killing of target species, but not subsequent meat-eating. Includes vocalizations such as *bark* or *scream*.
Invitation-sexual	Includes sexual presenting by females in oestrus (non-oestrus females are considered to be seeking affiliation when presenting genitals, as are males). Also includes behaviour relating to the inspection of the female swelling and male-female mounting and copulation from all age groups. Includes gestures such as *Object shake* or *Leaf clip*, and vocalizations such as *scream* or *pant*.
Intercommunity encounter^a^	Includes group patrolling (individuals walk one behind each other in a line while remaining silent and highly vigilant) towards location of a neighbouring community, or individual joining the group in an existing encounter. Includes gestures such as *Object shake* or *Object hit*, and vocalizations such as *scream* or *pant-roar*.
Moving canopy	Locomotion from one area to another within the canopy. Does not include brief locomotion between individuals within a group. Gestural signals rare but includes, for example, *Stomp object* and vocalizations such as *pant-hoot*.
Moving up/down tree	Climbing up or down between the ground and the canopy. Gestural signals rare, vocalizations such as *pant-hoot* or *food-grunt*.
Play-social	Two or more individuals engaged in play behaviour, may include both chasing-play and/or contact-play such as wrestling. Gestural signals common and varied, for example: *Object in mouth* or *Dangle*, and vocalizations such as *pant* or *laughter*.
Resting	An individual remains stationary without participating in any self-directed or other-directed physical activity; includes sleeping. Communication rare but includes gestures such as *Directed push* or vocalizations such as *hoo*.
Travel	Locomotion from one area to another on the ground. Does not include brief locomotion between individuals within a group. Includes gestures such as *Drum object* and *Arm swing* or vocalizations such as *hoo* or *pant-hoot*.

^a^Data too few for analysis

^b^Not seen during data collection period;

^c^We define ‘Display’ following Nishida et al. 2010, p. 17

#### Snare-injured individuals

As any physical disability may influence the choice of communication channel or signal type, we included only individuals with no major injuries (i.e. at the level of the whole hand, foot or limb, rather than individual fingers or toes).

### Statistical analyses

Statistical analyses were carried out in SPSS Statistics, with means ± standard deviation given throughout. All data were tested for normality before statistical analysis. In some cases, data were non-normal but transformations could be applied. If this was the case, analyses employing transformed data are marked in the “Results” section. Individual data were transformed to a rate, typically as signals per hour, to remove any effect of pseudo-replication.

#### Specific transformations used

For skewness, *Z* values over 1.96 or under −1.96 were considered to be positively or negatively skewed, respectively. In the case of positive skew, a transformation of either √(*x*) was applied. In the case of negative skew, we transformed the data with √ ((*x*_max_ + 1) − *x*). If the transformed data conformed to requirements, parametric analyses were conducted, with post hoc *t* tests following a one-way ANOVA. In cases of non-homogeneous data, either non-parametric tests, such as chi-square tests, or parametric tests that take deviation from normality of equality of variance into account, such as ANOVA_Brown-Forsythe_, or *t* test_unequal variances_, were carried out and clearly marked.

If planned comparisons could be made, standard *t* tests or their non-parametric equivalents were used, with Bonferroni correction if the number of planned comparisons equalled or exceeded the number of experimental conditions. In the case of unplanned, post hoc tests, we used Tukey’s HSD (for equal sample sizes) or Games-Howell tests (sample sizes varied between conditions; requirement for homogeneity of variance was violated).

As ranking data were only available for mature male individuals, we analysed this separately from our analyses of age and sex. When investigating the effect of rank, we calculated a rate per minute of signal use within each context for each of the nine mature males, and examined how both rank (as a covariate) and context (as an independent factor) affected rate of signal use (within the eight contexts in which at least five of the nine males were recorded signalling: agonism, affiliation, display, feeding, grooming, moving in tree, resting and travelling). The error variance differed across the groups so we employed a Generalized Linear Model (Gamma with log link distribution), which is not affected by this, to test for change in the rate per minute of signal type production with change in male rank while controlling for context.

## Results

Party size ranged from 1 to 30 individuals, and mean party size varied from 10 to 14 individuals throughout the day (mean am: 9.7 ± 6.9, mid: 11.5 ± 7.7, pm: 13.8 ± 8.3). The rate of an individual’s signalling was consistent throughout the day (ESM, Table S1); we therefore combined data from across time periods in all subsequent analyses.

In total, we recorded 7076 vocalizations and 2372 gestures, which were part of 7756 signals given over the total observation period of 93 focal hours. Focal individuals produced 643 vocalizations and 671 gestures, within 1017 signals (547 vocal, 388 gestural, 82 combination) and, as potential recipients, were exposed to a total of 6433 vocalizations and 1701 gestures, within 6739 signals (5331 vocal, 968 gestural and 440 combination; Table 1).

### Production and exposure

As signallers, individuals differed in how they allocated their communication efforts to the different modalities (*f* = 30.5, *df* = 1.5, 42.6, *p* < 0.0001; repeated measures ANOVA_Greenhouse-Geisser_). This variation was not due to differences between vocal and gestural signalling (vocal: 5.8 ± 2.9 signals per hour (signals/h); gestural: 3.9 ± 3.2 signals/h; mean difference = 1.9, *p* = 0.07; post hoc test with Bonferroni correction), but related to differences between single vocal or gestural signalling, and combination signalling (combination: 0.9 ± 1.0 signals/h; vocal vs. combination: mean difference = 4.9, *p* < 0.0001; gestural vs. combination: mean difference = 3.0, *p* < 0.0001).

Potential recipients were exposed to vocal signals (56.1 ± 16.6 signals/h) significantly more often than to gestural signals (10.4 ± 5.1 signals/h) or to combination signals (4.7 ± 2.4 signals/h; *f* = 289.7, *df* = 1.2, 34.1, *p* < 0.0001 repeated measures ANOVA_Greenhouse-Geisser_; post hoc tests with Bonferroni correction: mean difference vocal and gestural = 45.7, *p* < 0.0001; mean difference vocal and combination = 51.4 *p* < 0.0001).

Signallers produced an average 9.8 ± 4.8 signals/h. Single units were more frequent than series (single: 7.7 ± 3.2 per hour; series: 2.1 ± 1.9 per hour; *t* = 14.12, *df* = 29, *p* < 0.0001; paired *t* test, two-tailed). There was no difference between the frequencies of single type (gestural or vocal) or combination series (single type signals: 1.4 ± 2.3 per hour; combination series: 0.9 ± 1.0 per hour; *t* = 1.36, *df* = 29, *p* = 0.185; paired *t* test, two-tailed). Series typically consisted of two units, irrespective of modality (gestural: mean produced = 2.5 ± 1.0, range 2–9 gesture types; vocal: mean produced = 2.0 ± 0.2, range 2–3 call types; combination: mean produced = 2.3 ± 0.7, range 2–5 units). Combination series typically started with a vocalization (data produced and received by focal individuals combined: vocalization first = 71, gesture first = 11, chi-square = 43.0, *df* = 1, *p* < 0.0001). If three or more units were combined, they typically consisted of single vocalizations combined with two or more gestures (≥3 units: *n* = 37, all combinations); in contrast, single gestures combined with two or more vocalizations were less common (*n* = 16; chi-square = 8.3, *df* = 1, *p* = 0.004, see ESM Table S2).

### Sex and age

We found no effect of sex on the rate with which chimpanzees employed the different signal types during communication (gestural, vocal or combination signals; repeated measures ANOVA_Greenhouse-Geisser_: *F* = 0.079, *df* = 1.5, *p* = 0.880), but there were strong effects of age.

**Fig. 1:**
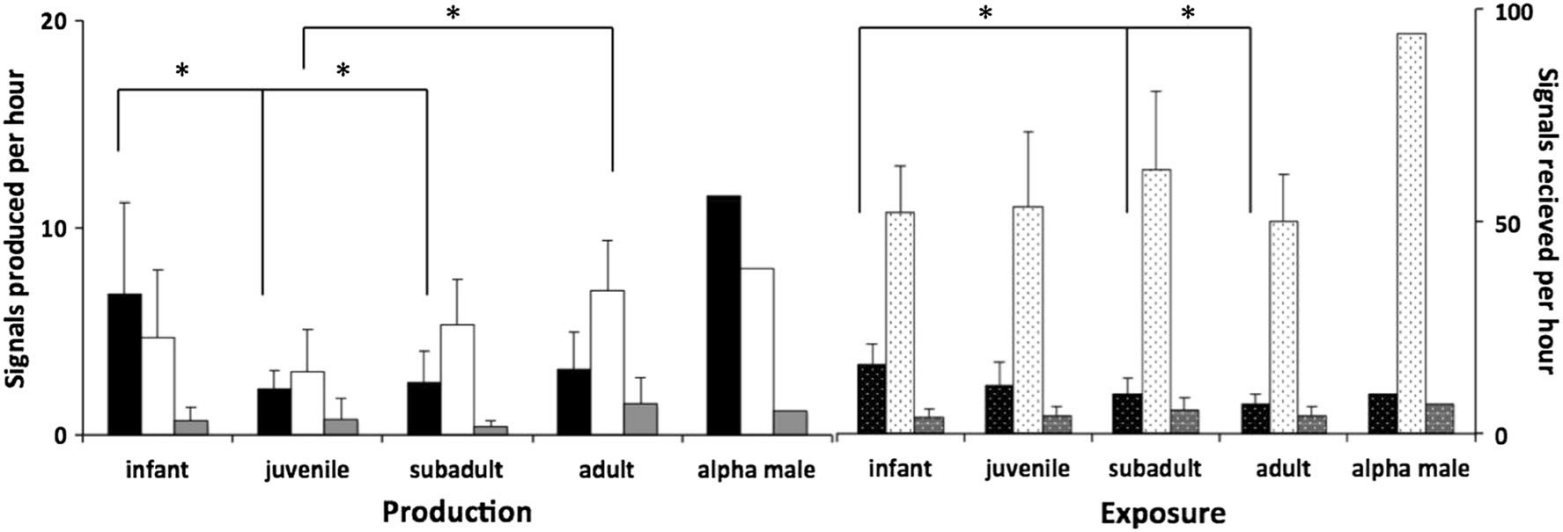
Mean production and exposure rate of signals for age categories and alpha male. Production shown on *left*: *black*, *white* and *grey bars* represent gestural, vocal and combination signals, respectively. *Error bars* show SD. Exposure shown on *right*: *bars spotted* to differentiate from production. Note different scales for production and exposure. *Asterisk* represents *p* < 0.05

Rates of gestural signals varied significantly across age groups (data transformed to correct for positive skew with √(*x*): one-way ANOVA *F* = 3.95, *df* = 3, 28, *p* = 0.020). Infants employed gestural signals more often than juvenile and subadults (infants = 6.8 ± 4.5 signals per hour (signals/h); juveniles = 2.2 ± 0.9 signals/h, post hoc Tukey HSD: *p* = 0.025; subadults = 2.6 ± 1.5 signals/h, post hoc Tukey HSD: *p* = 0.029), but not significantly more often than adults (mean rate = 3.2 ± 1.8 signals/h, post hoc Tukey HSD: *p* = 0.111; Fig. 1). Rates of vocal signals also varied between age groups (one-way ANOVA *F* = 3.22, df = 3, 25, *p* = 0.040) although in a different way. Aside from a minor peak in infancy (mean rate = 4.7 ± 3.2 signals/h), there was a significant rise in use of vocalizations with increasing age from juveniles to adults (post hoc Tukey HSD; *p* = 0.025; juveniles = 3.0 ± 0.9 signals/h; adults = 7.0 ± 2.5 signals/h). Rates of combination signals, finally, did not vary between age groups (data transformed to correct for positive skew with √(*x*): one-way ANOVA *F* = 1.77, *df* = 3, 28, *p* = 0.179; Fig. 1), with use at a mean rate of 0.83 ± 1.0 signals/h.

Taking the perspective of the potential recipients, we found no difference in the rates at which the different age groups were exposed to vocal or combination signals (vocal: *F* = 0.93, *df* = 3, 25, *p* = 0.44; combination: *F* = 0.62, *df* = 3, 35, *p* = 0.61, one-way ANOVAs). The rate at which individuals were exposed to gestural signals, however, varied across age groups with a general decrease as a function of age (*F* = 5.27, *df* = 3, 25, *p* = 0.006; one-way ANOVA; infants = 16.4 ± 4.8 signals/h; juveniles = 11.4 ± 5.6 signals/h; subadults = 9.3 ± 3.9 signals/h; infants vs. juveniles: *p* = 0.199; infants vs. subadults: *p* = 0.034; infants vs. adults: *p* = 0.004; post hoc Tukey HSD).

### Male rank

Mature males, including all adult and subadult individuals, were assigned a linear rank from 1 to 9. The alpha male’s production of gestural signals was more than 4 standard deviations above the adult mean (Fig. 1), and also higher than that of infants. As a recipient, the alpha male’s exposure to vocal and combination signals was also well outside of the normal range (vocal: alpha = 94.3 signals/h, adult mean = 50.1 ± 10.8 signals/h; combination: alpha = 7.2 signals/h, adult mean = 4.3 ± 2.2 signals/h; Fig. 1). However, the alpha male’s behaviour during the day also varied from other individuals—for example he was recorded in the context of ‘display’ more often than other subadult and adult males (8.3 min/h in contrast to 1.2 min/h; *n* = 8, range 0.3–2.3 min/h). We performed a generalized linear model for each signal type (fit statistics were good for all three models: *Χ* ^2^/*df*: gestural = 1.09; vocal = 1.42; combination = 1.04). When controlling for context, we found no effect of rank on the rate per minute of either vocal or combination signals in male chimpanzees, and only a very small increase in gesture rate per minute with increase in male rank (see Table 3). Given the special status of the alpha male in the chimpanzee hierarchy, we re-ran the same models after excluding the alpha male (*n* = 8 individuals, fit statistics were again good for all three models: *Χ* ^2^/*df*: gestural = 0.43; vocal = 1.27; combination = 0.92). When controlling for context, we again found no effect of rank on the rate per minute of vocal signals in (non-alpha) male chimpanzees. However, with the alpha male excluded, we found no effect of (non-alpha) male rank on the rate of gestural signals and a small increase in the rate per minute of combination signals with increase in (non-alpha) male rank (see Table S4), suggesting that the alpha male may have been driving the effect of rank on gesture, and masking a small effect of rank on the use of combinations.

**Table 3:** Effect of male rank on frequency of signal use while controlling for context. Generalized linear model: negative value of *B* together with *p* < 0.05 indicates increase in rate of signal use with increase in rank. All adult and subadult males included, *n* = 9

Signal type (rate per min)	*B* (SE)	95% Wald confidence interval for (*B*)		Wald chi-square (*df*)	*p* value
		Lower	Upper		
Gestural	−0.10 (0.04)	−0.19	−0.02	5.69 (1)	0.017
Vocal	0.09 (0.07)	−0.05	0.23	1.76 (1)	0.184
Combination	−0.08 (0.05)	−0.19	0.02	2.33 (1)	0.127

### Persistence

**Fig. 2:**
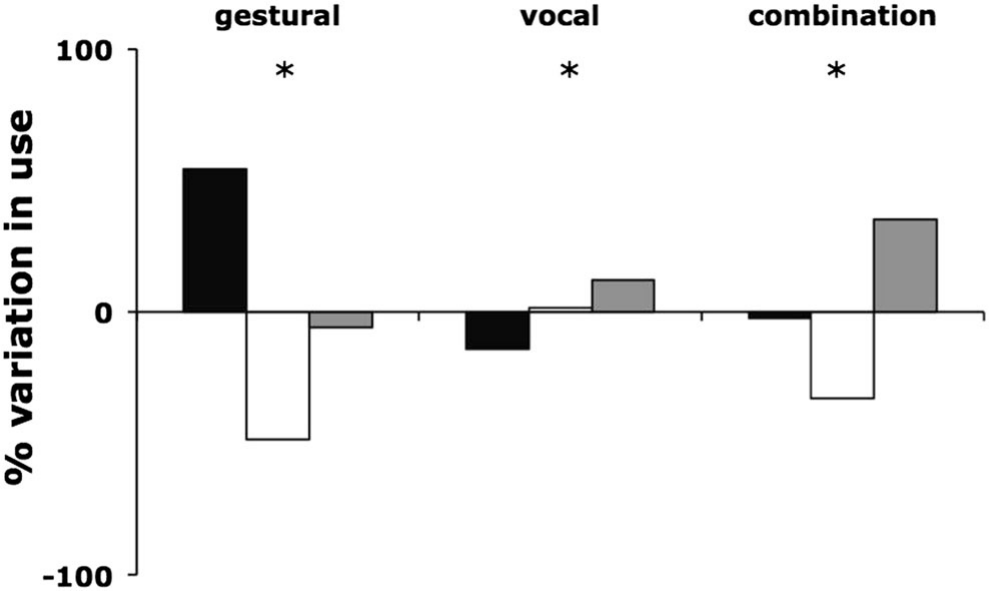
Percentage variation in distribution of modalities during persistence following use of each of the three modalities. Zero represents expected normal distribution with which modalities occur in whole data set; variation from this marked in percentage frequency of use. *Asterisk* represents significant variation from the norm (*p* < 0.001). *Black*, *white* and *grey bars* represent respectively gestural, vocal and combination signals produced after response waiting

We compared the use of vocal, gestural, and combination signals following an apparently failed signal with the general distribution of these signal types in communication. Following gestural signals, individuals were more likely to produce further gestural signals (chi-square = 121.4, *df* = 2, *p* < 0.00001). Similarly, following combination signals, signallers were more likely to produce further combination signals (chi-square = 23.0, *df* = 2, *p* < 0.00001). Following vocal signals, however, signallers were more likely to produce combination signals (chi-square = 44.7, *df* = 2, *p* < 0.00001; Fig. 2).

### Variation in use across contexts

We compared the distribution of signal modalities produced by focal individuals in 15 behavioural contexts with the distribution of signal types in the data set as a whole across all contexts. Of the 15 contexts recorded, 4 contexts (Begging, Displace, Hunting and Intercommunity encounter) contained too few data for analysis. If chimpanzees are sensitive to the distinction between public and private signalling, then during displays or sexual invitations, when male signallers would incur a low benefit and potentially high cost from the use of signals that revealed their identity to out-of-sight individuals, they should employ a relatively greater proportion of gestural communication when compared with their use across all other contexts. Consistent with this prediction, during displays and sexual invitations the proportion of gestural signals in individual signaller’s communication nearly tripled (paired *t* test; *p* < 0.0001 *n* = 12 signallers; gestural proportion displays and sexual invitations = 0.85 ± 0.23; gestural proportion all other contexts = 0.29 ± 0.23) and the proportion of vocal signals decreased (paired *t* test; *p* < 0.0001 *n* = 12 signallers; vocal proportion displays and sexual invitations = 0.04 ± 0.14; vocal proportion all other contexts = 0.60 ± 0.21) but the use of signal combinations did not change (paired *t* test; *p* = 0.948 *n* = 12 signallers; combination proportion displays and sexual invitations = 0.11 ± 0.17; combination proportion all other contexts = 0.11 ± 0.09). In contrast, when travelling, moving in trees or resting, signallers would potentially incur a low cost and/or high benefit to transmitting their identity to out-of-sight individuals and may employ a greater proportion of vocal communication. Consistent with a sensitivity to the public/private nature of information, the proportion of vocal signals in their communication increased when travelling, moving or resting (paired *t* test; *p* < 0.0001, *n* = 30 signallers; vocal proportion of travelling, moving and resting communications = 0.71 ± 0.18; vocal proportion of all other contexts = 0.37 ± 0.25) and the proportion of gestural communication decreased (paired *t* test; *p* < 0.0001, *n* = 30 signallers; gestural proportion travelling, moving and resting communications = 0.22 ± 0.19; gestural proportion of all other contexts = 0.54 ± 0.29) but, again, their use of signal combinations did not change (paired *t* test; *p* = 0.400, *n* = 30 signallers; combination proportion of travelling, moving and resting communications = 0.07 ± 0.08; combination proportion of all other contexts = 0.09 ± 0.10).

**Fig. 3:**
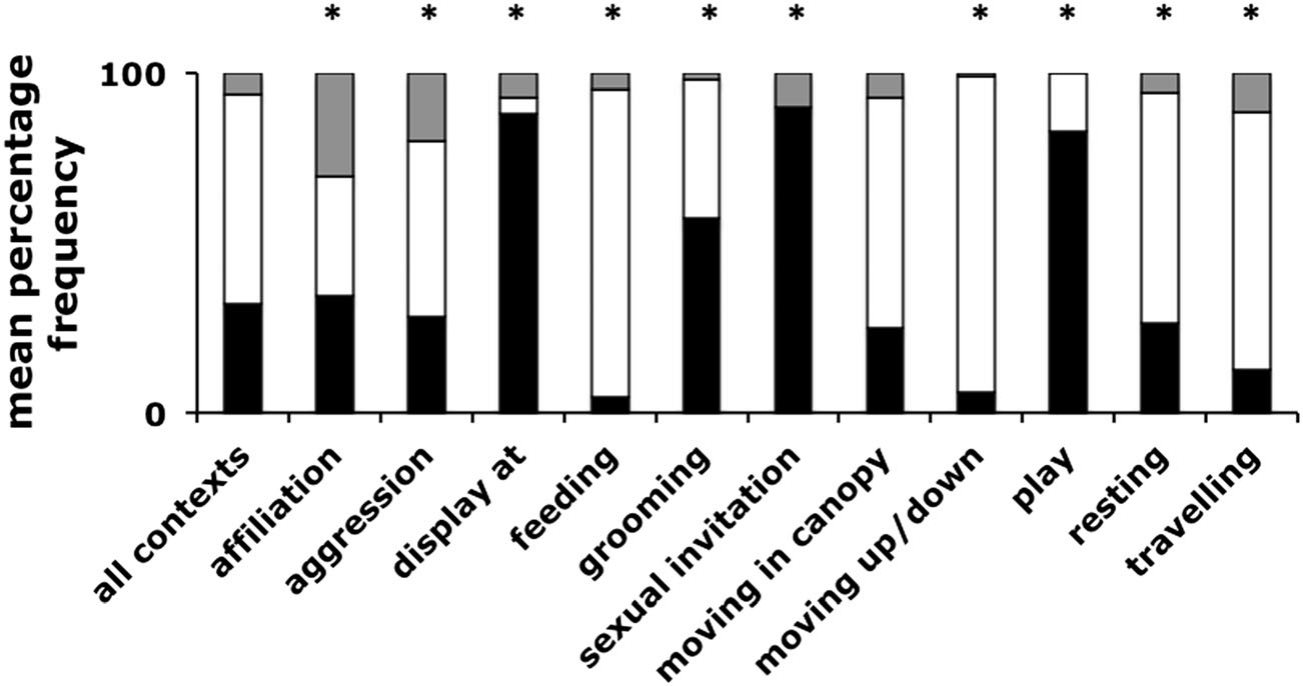
Distribution of signal modalities within behavioural context (15 or more cases). Values calculated by averaging across individual means. *Black*, *white* and *grey segments* represent gestural, vocal and combination signals, respectively. *Asterisk* indicates distribution deviated significantly from normal distribution (calculated from all contexts)

Within the remaining five contexts (affiliation, agonism, feeding, grooming and play), signallers could accrue either costs or benefits depending on their role (for example: in an aggressive attack where an out-of-sight individual was allied to the victim, an individual in the attacking role may accrue a cost to revealing their identity, while an individual in the victim role may accrue a benefit). To investigate signal use, we conducted chi-square goodness of fit tests, comparing the observed distribution of signal types with the expected distribution (the distribution of signal types within the total data across all contexts). All five contexts varied from the expected distribution, the strongest bias towards combination signalling was observed during affiliative and agonistic contexts, at the expense of vocal signalling, which decreased relative to its use across all contexts (Fig. 3, Table S3). Grooming and play showed increased use of gestural signals, whereas within feeding there was an increase in vocal signals.

## Discussion

In animal communication, including human language, a single signal can be multimodal if it combines visual, audible and tactile elements. Here we consider an additional level of potential flexibility: the combination of two different types of signals, gestures and vocalizations. Our data support the finding that chimpanzees employ vocal and gestural signals both separately and in combination, suggesting that the combination of gestural and vocal signals as seen in human language is not unique to the human lineage. Moreover, they adjust their use of signal types depending on social context and success of previous communications.

Chimpanzees have been argued to be largely vocal communicators, in consequence of the dense, visually obscured, nature of their forest habitat (Slocombe and Zuberbühler 2009). However, in this study we find that chimpanzees employ both vocal and gestural signals with equal frequency. Certainly, given their dense habitat that allows the receipt of vocalizations but not gestures from out-of-sight individuals, the communicative world that they are exposed to is a highly vocal one. Gestural communication tends to be focused on immediate targets in specific contexts, whereas vocal signals may be received from multiple signallers in and out of visual range—highly adaptive in a fission-fusion society, but not without potential cost depending on the information encoded within a particular signal. We found that combination signals were rare and, as recorded in chimpanzees (Leavens and Hopkins 2005; Pollick and de Waal 2007; Leavens et al. 2010; Wilke et al. 2017) and bonobos (Pollick and de Waal 2007; Pollick et al. 2008; Genty et al. 2014), were employed less frequently than either gestures or vocalizations in isolation. Combinations were mainly used in complex social interactions, as seen in affiliative and agonistic behaviour, and especially after a failure of vocal signals.

The alpha male appeared to be an outlier both as a signaller and recipient: an extremely prolific user of gestures and exposed to particularly high levels of vocal and gesture-vocal signals. When we controlled for the context in which the communication occurred, we found a small positive effect of male rank on gesture use. However, this effect disappeared when the alpha male was excluded suggesting that prolific gesture use may be a feature of the unique position the alpha male occupies rather than of higher-ranks in general, and it may be the result of his spending more time in contexts particularly associated with gesture use, such as display. The similarly prolific production of gesture in infants may also be influenced by context, in this case most likely that of play. However, another possible explanation is that infants tend to limit their communication to individuals in close proximity to them, for example their mother and siblings, which may favour use of gesture. With the alpha male excluded, we found a small increase in the use of combination signals with an increase in male rank.

Distinguishing different pressures that have driven the evolution of signal combinations is not straightforward, particularly as several pressures may interact on the same system, such as increased efficacy, and modification or refinement of content (Wilson et al. 2013). Addressing this issue will require detailed study of recipient behaviour, and its effect on choice of gestures, vocalizations and their combinations (Hasson 1994; Maynard Smith and Harper 1995; Genty et al. 2009; Hobaiter and Byrne 2014; Wilke et al. 2017) as well as distinguishing among different gestural and vocal signals and taking into account communication efforts over long and short distances. However, the patterns of behaviour in the selection of signal types offer some indications.

### Redundancy versus refinement

Great apes regularly produce series of signals. In gestural communication, this includes the rapid combination of signals in a sequence independently of recipient behaviour, perhaps as a ‘fail safe’ strategy (Hobaiter and Byrne 2011b), and also the addition of further signals following response waiting that indicates persistence towards an intended goal following the failure of earlier signals in the series (Liebal et al. 2004, Hobaiter and Byrne 2011b, Roberts et al. 2013). If the use of gestural and vocal signal combinations reflected a strategy of redundancy to improve signalling efficacy (the additional signal type being added to improve transmission success), we would expect that signal combinations would follow on from the failure of the single use of either a gestural or vocal signal. However, only if their initial signals were vocal were signallers likely to switch from single to combined signal use after failed communication efforts; not if they were gestural. This difference in patterns of persistence depending on signal type suggests that there may be variation in the type of information encoded in vocal and gestural signals. If both signal types encoded similar information, their combination may provide redundancy; however, differences in the type of information encoded suggest that their combination might allow for refinement or modification of content. Similarly, Wilke et al. (2017) recently found evidence that chimpanzee signal combinations (facial, vocal or gestural) were more likely to elicit a behavioural response than vocal signals alone, but not more likely to do so than gestural signals alone, and suggested that the vocal signals may serve as an attention getter, or to disambiguate the signaller’s meaning.

### Private versus public messaging

Chimpanzees employed signal combinations most often in the contexts of affiliation and agonism, where misinterpretation of signals may be particularly costly. The potential costs of miscommunication in an agonistic context are immediately evident, with serious injury and even lethal aggression present between group members (Wrangham et al. 2006; Wilson et al. 2014), but affiliative contexts are also important. In chimpanzee society, the subtle regulation of individual relationships is important for the formation and maintenance of long-term social bonds, which impact social and reproductive success (e.g. Duffy et al. 2007; Gomes and Boesch 2009; Gilby et al. 2013). Indeed, in other primate communication systems, it is the degree of social tolerance that increases signal complexity and flexibility (Maestripieri 1999; Dobson 2012), and in chimpanzee gestural communication an increased range of signals is employed for ‘social negotiation’ in the subtle regulation of complex social relationships (Hobaiter and Byrne 2014).

Signal selection can vary with context for purely practical reasons. When climbing, limbs are employed in locomotion and may be unavailable for gesturing, and indeed here the use of vocal signals was increased. Similarly, signal selection may be affected by environmental constraints. Around a third of gestural signals have an individually distinctive audible component (Hobaiter and Byrne 2011a; for example, a *Leaf clip* or *Branch shake*), and may transmit this information over a similar range to short-distance vocalizations (for example, a *Pant-grunt* or *Travel-hoo*). Nevertheless, their acoustic properties are quite different to vocalizations, and so some vocal signals may be more effective within contexts that require more frequent communication with out-of-sight individuals. The very graded nature of the vocal repertoire also allows chimpanzees to produce a very wide range of call variations; for example, a *Pant-grunt* may be produced alone or combined with a clear *Bark* call, but in addition a range of intermediate calls may be produced where the grunt becomes increasingly ‘bark-like’. However, signal selection may also offer the opportunity to discriminate between the information provided to different audiences, particularly where eavesdroppers may gain valuable information (Wilson et al. 2013; Fedurek et al. 2015). In any one context, the cost and benefit of revealing one’s identity to out-of-sight individuals may depend on role. For example, it would pay an attacker to conceal its identity from potential out-of-sight allies of its victim, while the victim would benefit from broadcasting its identity and the information that it is being attacked. However, in some behavioural contexts, clearer predictions can be made. When soliciting sexual attention from a female or engaging in dominance displays, male chimpanzees may suffer high cost from revealing their identity to out-of-sight individuals. Conversely, there is potential benefit to communicating individual location to out-of-sight others when travelling or resting. We find that within these contexts individual chimpanzees employ their signals in accordance with a cost/benefit analysis: increasing their use of gesture in contexts where eavesdropping imposes high costs, and increasing their use of vocalizations in contexts in which they may incur benefits from revealing their identity widely.

Understanding how and when gestures and vocalizations are employed either singly and in combination is important to discriminating between hypotheses of different selective pressures in the evolution of more complex, more language-like communication. As Slocombe et al. (2011) suggested, it is critical that comparative communication research address not only questions of the integration of information from different channels into a single signal, but specifically address the combination of gestural and vocal signals. By considering the full range of gestural and vocal signals available to them, we demonstrate that wild chimpanzees adjust their signal selection depending on the success of previous signal types, and according to whether or not their audiences are within visible range. The combined use of vocal and gestural signals may also offer increased subtlety during complex social interactions. We suggest that in future studies it is crucial to consider both the signal type: gesture, vocalization and the different modalities of information: audible, silent visual, tactile, encoded within primate communication.

## Electronic supplementary material

ESM 1 (DOCX 99 kb)
