# Supplementary material for: Wild chimpanzees’ use of single and combined vocal and gestural signals
Source: Behav Ecol Sociobiol. 2017 May 27;71(6):96. doi: 10.1007/s00265-017-2325-1 (PMC5446553; doi:10.1007/s00265-017-2325-1)
Supplement: Supplementary file 1 — (DOCX 99 kb) [file 265_2017_2325_MOESM1_ESM.docx]

Wild chimpanzees’ use of single and combined vocal and gestural signals

Behavioural Ecology and Sociobiology

C. Hobaiter^* a,b^, R.W. Byrne^a^, K. Zuberbühler^a,b,c^

^a^School of Psychology and Neuroscience, University of St Andrews, Scotland

^b^Budongo Conservation Field Station, Masindi, Uganda

^c^Department of Comparative Cognition, University of Neuchatel, Switzerland

^*^Corresponding author: [clh42@st-andrews.ac.uk](mailto:rwb@st-andrews.ac.uk)

**ESM Results**

The rate of an individual’s signalling was consistent throughout the day (table S1); (Repeated measures ANOVAs, n=30 individuals: F=0.675, df=2, p=0.513; gestural (g) signals: F=0.219, df=2, p=0.804; vocal (v) signals: F=1.15, df=2, p=0.325; multimodal (mm) signals: F=0.14, df=2, p=0.869). We therefore combined data from across time periods in all subsequent analyses.

**table S1: Frequency of communication modalities with time of day.** Numbers represent signals produced by the focal individuals; numbers in brackets represent signals received by the focal individuals.

| **Signal modality** | **Time of day** | | | **All**  **total** |
| --- | --- | --- | --- | --- |
|  | **AM total** | **MID total** | **PM total** |  |
| Gestural | 119 (282) | 140 (294) | 129 (392) | 388 (968) |
| Vocal | 152 (1690) | 204 (1640) | 191 (2001) | 547 (5331) |
| Multimodal | 29 (151) | 24 (118) | 29 (171) | 82 (440) |
| **All** | **300 (2123)** | **368 (2052)** | **349 (2564)** | **1017 (6739)** |

***Signal combinations***

Signallers typically combined just two units into a series (gestural: mean produced=2.5±1.0 range 2-9 gesture types; vocal: mean produced=2.0±0.2 range 2-3 call types; multimodal: produced by focal: mean=2.3±0.7, range 2-5 units). Similarly as recipients of series, focal animals predominantly received two-unit series regardless of modality (gestural: mean=2.3±0.6, range= 2-7 gesture types, vocal: mean=2.1±0.4, range 2-3 call types; multimodal: mean=2.1±0.4, range 2-5; see table S2). When receiving multimodal series, these typically started with a vocalization (mm series received by focal: vocalization first =413, gesture first = 27, chi-square =338.6, df=1, p<0.0001).

**table S2: multimodal combinations.** Number of vocal and gestural units in multimodal combinations, all data (produced and received by focal individuals) combined.

| n gestural units | n vocal units | | | |
| --- | --- | --- | --- | --- |
|  | **1** | **2** | **3** | **4** |
| **1** | 464 | 16 | 0 | 0 |
| **2** | 23 | 2 | 0 | 0 |
| **3** | 10 | 0 | 0 | 0 |
| **4** | 4 | 0 | 0 | 0 |

**Table S3: distribution of signal types in each behavioural context.** Values (n) are the sum total from all focal individuals; mean percentage frequency (%) is calculated by averaging across individuals.

|  | **Signal modality n (mean percentage frequency)** | | | |
| --- | --- | --- | --- | --- |
| **Context** | **Gestural**  **n (%)** | **Vocal**  **n (%)** | **Combination n (%)** | **Chi-square (p value)** |
| Affiliation | 15 (34) | 17 (35) | 8 (31) | 0.029 |
| Aggression | 8 (28) | 21 (52) | 14 (20) | 0.000 |
| Feeding | 6 (5) | 61 (90) | 1 (5) | 0.000 |
| Grooming | 31 (57) | 17 (41) | 2 (2) | 0.005 |
| Play | 112 (83) | 19 (17) | 0 (2) | 0.000 |
| All contexts | 376 (32) | 494 (62) | 80 (6) |  |

**Table S4: Effect of male rank in non-alpha mature male chimpanzees on frequency of signal use while controlling for context.** Generalized linear model: negative value of B together with p<0.05 indicates increase in rate of signal use with increase in rank. All adult and sub-adult males except the alpha male included, n=8.

| **Signal type (rate per min)** | **B (S.E.)** | **95% Wald confidence interval for (B)** | | **Wald Chi-square (df)** | **p** |
| --- | --- | --- | --- | --- | --- |
|  |  | Lower | Upper |  |  |
| Gestural | -0.02 (0.04) | -0.09 | 0.06 | 0.18 (1) | 0.672 |
| Vocal | 0.05 (0.09) | -0.13 | 0.22 | 0.27 (1) | 0.602 |
| Combination | -0.17 (0.06) | -0.30 | -0.05 | 7.89 (1) | 0.005 |
